# Comparing BMI with skinfolds to estimate age at adiposity rebound and its associations with cardio-metabolic risk markers in adolescence

**DOI:** 10.1038/s41366-018-0144-8

**Published:** 2018-07-13

**Authors:** Chiara Di Gravio, G. V. Krishnaveni, R. Somashekara, S. R. Veena, K. Kumaran, Murali Krishna, S. C. Karat, Caroline H. D. Fall

**Affiliations:** 1 MRC Lifecourse Epidemiology Unit, University of Southampton, Southampton, UK; 2 CSI Holdsworth Memorial Hospital, Mysore, India

**Keywords:** Epidemiology, Risk factors

## Abstract

**Background:**

Body mass index (BMI) reaches a nadir in mid-childhood, known as the adiposity rebound (AR). Earlier AR is associated with a higher risk of cardio-vascular diseases in later life. Skinfolds, which are a more direct measure of adiposity, may give better insight into the relationship between childhood adiposity and later obesity and cardio-metabolic risk.

**Objective:**

We aimed to assess whether AR corresponds to a rebound in skinfolds, and compare associations of BMI-derived AR and skinfold-derived AR with cardio-metabolic risk markers in adolescence.

**Methods:**

We used penalised splines with random coefficients to estimate BMI and skinfold trajectories of 604 children from the Mysore Parthenon Birth Cohort. Age at AR was identified using differentiation of the BMI and skinfold growth curves between 2 and 10 years of age. At 13.5 years, we measured blood pressure, and glucose, insulin and lipid concentrations.

**Results:**

BMI and skinfolds had different growth patterns. Boys reached BMI-derived AR earlier than skinfold-derived AR (estimated difference: 0.41 years; 95% CI:[0.23, 0.56]), whereas the opposite was observed in girls (estimated difference: −0.71 years; 95% CI:[−0.90, −0.54]). At 13.5 years, children with earlier BMI-derived AR had higher BMI (−0.58 SD per SD increase of AR; 95%CI:[−0.65, −0.52]), fat mass (−0.44; 95%CI:[−0.50, −0.37]), insulin resistance (HOMA-IR: −0.20; 95%CI:[−0.28, −0.12]) and systolic blood pressure (−0.20; 95%CI:[−0.28, −0.11]), and lower HDL-cholesterol (0.12; 95%CI:[0.04, 0.21]). The associations were independent of BMI at time of rebound, but were fully explained by fat mass at 13.5 years. Similar associations were found for skinfold-derived AR.

**Conclusion:**

BMI-derived adiposity rebound predicts later cardio-metabolic risk markers similarly to that derived from skinfolds, a direct measure of adiposity.

## Introduction

Cardiovascular disease (CVD) is the primary cause of death in Indian adults, with age-standardised death rate of 348.9 per 100,000 men and 246.6 per 100,000 women [1]. The Global Burden of Disease Study estimated that, in 2015, CVD accounted for more than 25% of deaths in India [2]. Growth in early life is an important predictor of later CVD risk; studies in both high and low and middle income countries (LMICs) have shown that lower birth weight and greater childhood BMI gain are associated with an increased risk of CVD and its factors hypertension and Type 2 diabetes [3–6].

BMI is widely used as a proxy for adiposity due to its simplicity of measurement and low cost. Generally, BMI increases rapidly in the first two years of life, then decreases and reaches a nadir around 5–7 years, before increasing again [7]. The age corresponding to the lowest BMI value recorded before the onset of its second rise is called the adiposity rebound (AR) [8]. Earlier AR is associated with higher BMI in later childhood [9] and adulthood [7, 10], and a higher risk of Type 2 diabetes [11].

While BMI is highly correlated with adiposity [12–14], it is still unknown whether AR represents a true rebound in adiposity. Firstly, BMI includes both fat and lean mass. Studies in high income countries have shown that the age at BMI rebound does not correspond to a rebound in fat mass, but results from an increase in lean mass index combined with a stabilisation of fat mass index [15]. Secondly, BMI depends on both height and weight; hence, if height grows faster than weight, BMI can decrease even if adiposity (fatness) does not [16]. Taller children have an early AR [17] suggesting that AR reflects the relative rates of height and weight gain rather than changes in adiposity, and may indicate advanced maturation [18].

To our knowledge, no studies in LMICs have investigated whether the BMI-derived AR corresponds to a rebound in adiposity and compared their associations with later obesity and cardio-metabolic risk markers. In the present study, we used longitudinal height, weight and skinfold measures from a prospective cohort of children living in Mysore, India, to examine (1) whether there is a rebound in skinfold thickness, a direct measure of adiposity, (2) if so, whether it corresponds in timing to the BMI-derived AR and (3) how it compares with BMI-derived AR in its associations with obesity and cardio-metabolic risk markers measured at 13.5 years.

## Methods

Data were collected within the Mysore Parthenon Birth Cohort, a prospective cohort set up to examine the long-term effects of early life factors on later cardio-metabolic health. Detailed information on the cohort has been published elsewhere [19]. Briefly, between June 1997 and August 1998, 830 women attending antenatal clinics at CSI Holdsworth Memorial Hospital (HMH) in Mysore, India, with no known history of diabetes, having a singleton pregnancy and intending to deliver at HMH were recruited. Among those, 663 women delivered babies without major congenital abnormalities at HMH. Anthropometry (weight, crown-heel length, triceps and subscapular skinfold thickness) was recorded within 72 h from birth, and collected annually until 5 years of age and every 6 months after that. At each follow-up, triceps and subscapular skinfolds were measured in triplicate and subsequently averaged. Total skinfold thickness was calculated as the sum of the averages of triceps and subscapular skinfolds. Inter-observer coefficients of variation (CV) were low for both triceps and subscapular skinfolds ranging from 2.1% at birth to 8.2% at 5-year follow-up [20].

At 13.5 years, cardio-metabolic risk markers were measured and information on socio-economic status and pubertal stage was collected. Body fat was measured using bioimpedance (Bodystat, Quadscan 400, Isle of Man, UK) and its value was derived from the manufacturer’s equation. A comparison of body fat estimated from different published equations with measurements obtained using ^18^O dilution showed that, in a smaller sample of the present cohort, the manufacturer’s equation had the least bias [21]. Systolic and diastolic blood pressure were measured using an automated blood pressure monitor (Dinamap 8100, Criticon, FL, USA) after at least 5 minutes seated at rest. Fasting blood samples were used to assess plasma glucose, insulin and lipid concentration. Glucose and lipid concentrations were measured by standard enzymatic methods (Hitachi-902, Roche, Germany), while insulin was measured using an enzyme-linked immunosorbent assay (Mercodia Ultrasensitive, Mercodia AB, Uppsala, Sweden) [20]. Insulin resistance was estimated using the HOMA-IR equation [22]. Assays were carried out at the Diabetes Unit, KEM Hospital, Pune, India. Socio-economic status was assessed using the Standard of Living Index (SLI) estimated based on housing type, utilities and household possessions [23]. Pubertal stage was identified using Tanner’s method [24], considering the stage of breast development in girls and genital development in boys.

### Analysis sample

BMI and skinfold growth curves were derived using data from 604 children (a maximum of 14 values per child), after excluding children who died during follow-up (*n* = 25), those with major medical conditions (*n* = 8), and children with less than three records of BMI and/or skinfolds between 2 and 10 years of age (*n* = 26). Of the 604 children, only 5 (0.8%) did not have a rebound in BMI, while 109 (18%) had no skinfold rebound. We included these children in the analysis, and we hypothesised that they reached the rebound at 2 years of age as BMI/skinfolds had an upward trajectory after 2 years. Five-hundred and forty-five children were available for follow-up at 13.5 years, and were included in the analyses of associations between AR and cardio-metabolic risk markers.

### Statistical analysis

Sex-and-cohort-specific BMI and skinfold centile curves were derived using the Lambda–Mu–Sigma (LMS) method [25]. To estimate AR, subject-specific growth curves were fitted to BMI and skinfolds, respectively, using penalised splines with random coefficients. Briefly, penalised splines are piecewise polynomial curves connected at user-defined points (knots), subject to penalties ensuring smoothness, that model the relationship between variables without imposing any assumption on the trend (e.g. linear, quadratic, etc.) of the association with age [26]. Different numbers and positions of knots were used to choose the “best” model. Based on Akaike’s information criteria (AIC), five knots, placed at the quintiles of unique ages, were selected. BMI-derived age at AR and skinfold-derived age at AR were determined by differentiation of the corresponding fitted curves restricted to ages between 2 and 10 years [27].

Because BMI-derived age at AR and skinfold-derived age at AR were not normally distributed, Mann–Whitney *U* tests were used to look at the differences between sexes, and Wilcoxon signed rank tests were used to determine whether children reached minimum BMI and skinfolds at similar ages. Differences in means were reported as descriptive statistics together with 95% bootstrap confidence intervals. Bootstrap allows the derivation of the standard error and confidence interval for any estimator without making any assumptions about its distribution [28].

To understand the associations of BMI-derived and skinfold-derived age at AR with cardiometabolic risk markers at 13.5 years, we used a series of linear regression models adjusted for factors that are known to be associated with cardiometabolic risk markers at 13.5 years. Specifically, we first adjusted for age and sex (model 1); next, we further adjusted for height, socio-economic status, pubertal stage at 13.5 years and exposure to maternal gestational diabetes (model 2), and for BMI or skinfolds at the time of rebound (model 3). Finally, we further adjusted for fat mass at 13.5 years (model 4). For each cardio-metabolic outcome, we tested for interactions between sex and AR after adjusting for the child’s exact age at 13.5 years. To allow comparison between BMI-derived AR and skinfold-derived AR, outcome and exposure variables were transformed into standard deviation scores (SDS) for analysis and presentation.

The robustness of our findings was assessed through sensitivity analyses fitting the same set of models to a smaller sample obtained after removing (1) children who did not have a rebound in BMI or skinfolds between 2 and 10 years of age, (2) children with one or more BMI or skinfolds measure missing between 2 and 10 years of age. Results were considered statistically significant when *p* < 0.05. The analyses were performed using R V.3.4.1 [29].

### Ethics

The Holdsworth Memorial Hospital ethics review committee approved the study and informed written consent was obtained from the parents and assent from children.

## Results

### BMI and skinfold growth patterns

**Fig. 1:**
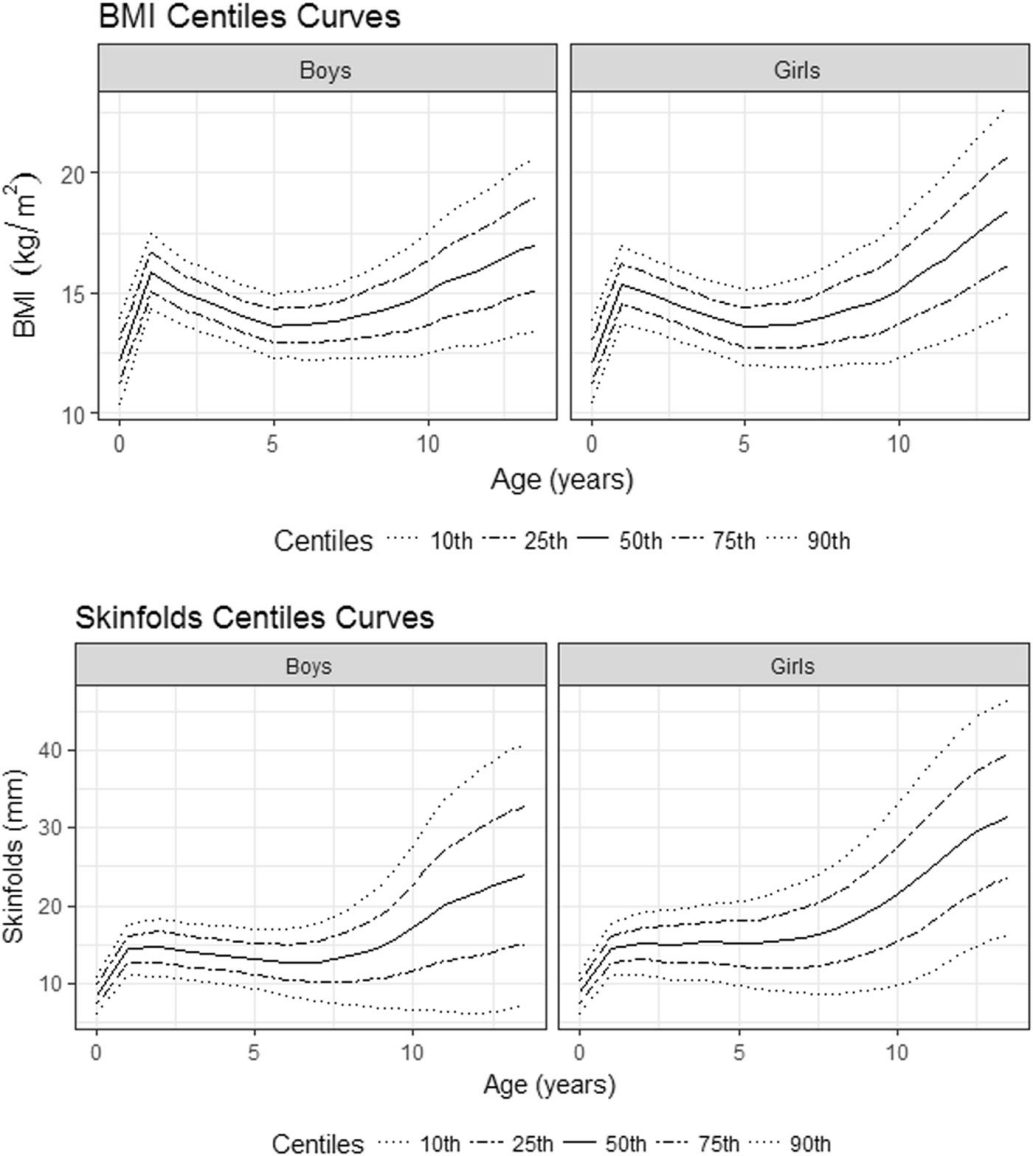
Smoothed reference curves for the 10th, 25th, 50th, 75th and 90th for BMI and skinfolds in 0–13.5 years old Indian boys and girls

BMI and skinfolds had similar growth patterns in boys and girls (Fig. 1). However, while BMI started to decrease at 2 years, and reached its second minimum after 5 years of age, skinfolds remained somewhat constant between 2 and 6 years and started increasing only after 6 years, with a steeper increase in girls. The different trends of BMI and skinfolds were reflected in the number of children who did not experience a rebound between 2 and 10 years of age.

**Fig. 2:**
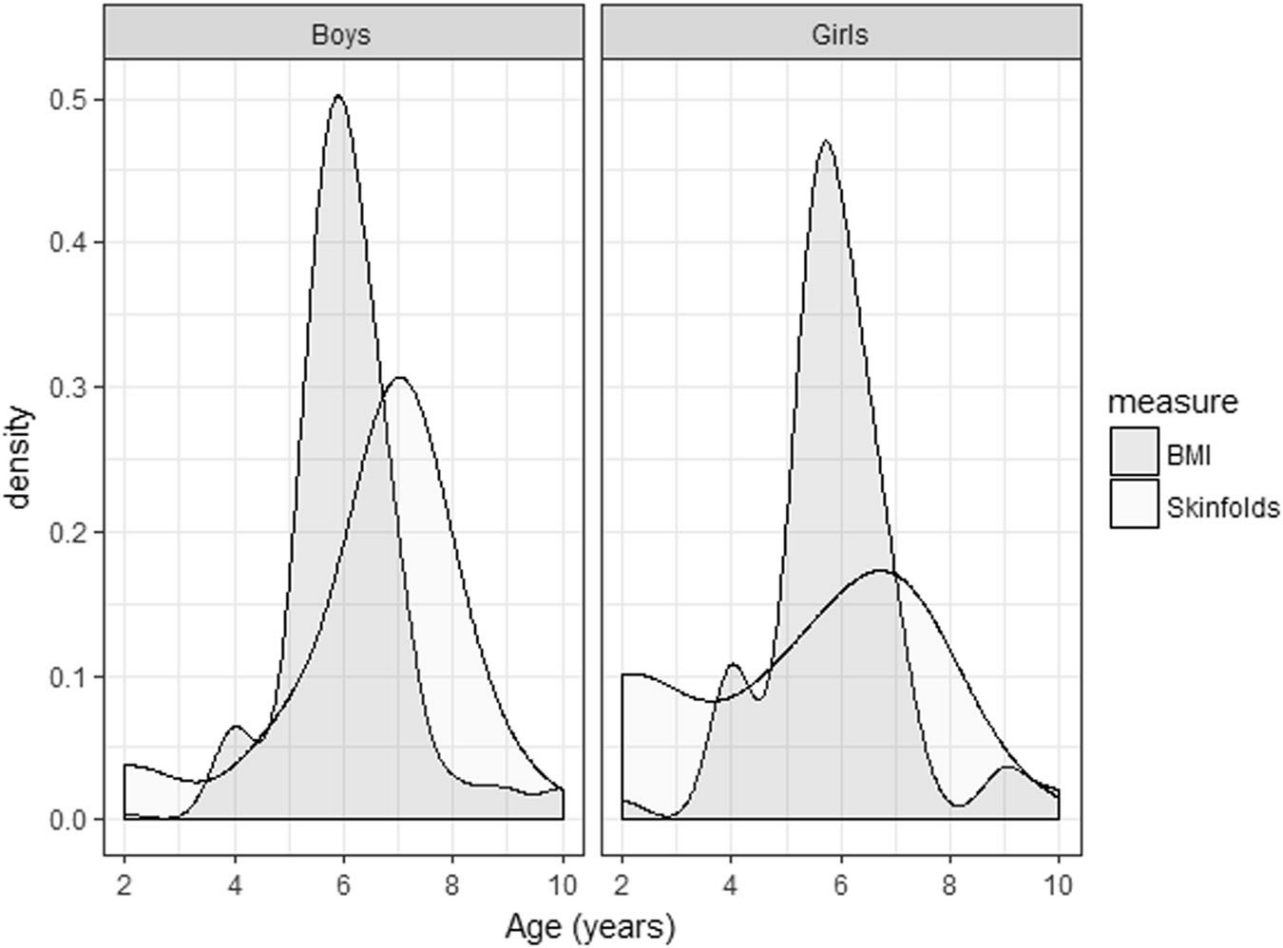
Distribution of BMI-derived adiposity rebound and skinfold-derived adiposity rebound by sex

Mean (SD) BMI-derived age at AR was 6.10 (1.08) years for boys and 5.90 (1.23) years for girls (estimated difference: −0.19 years; 95% CI: [−0.37, −0.01]; *p* = 0.01), whereas mean (SD) skinfold-derived age at AR was 6.51 (1.68) years for boys and 5.19 (2.23) years for girls (estimated difference: −1.31 years; 95% CI: [−1.61, −1.00]; *p* < 0.001). Skinfold-derived age at AR had greater variability than BMI-derived age at AR in both sexes (Fig. 2). Boys tended to reach skinfold-derived AR later than BMI-derived AR (estimated difference: 0.41 years; 95% CI: [0.23, 0.56]; *p* < 0.001), while the opposite was true for girls (estimated difference: −0.71 years; 95% CI: [−0.90, −0.54]; *p* < 0.001). Boys and girls had similar BMI at time of BMI-derived AR (estimated difference: −0.07 kg/m^2^; 95% CI: [−0.23, 0.09]; *p* = 0.21); however, girls had higher skinfold thickness at time of skinfold-derived AR (estimated difference 2.12 mm; 95% CI: [1.72, 2.54]; *p* < 0.001).

### Associations with later cardio-metabolic risk markers

Among the 545 children who were followed-up at 13.5 years, 48% were boys. Compared to girls, boys had lower BMI, fat mass, fasting insulin, triglycerides and HOMA-IR at 13.5 years but higher systolic and diastolic blood pressure (Table 1).

**Table 1:** BMI, fat mass and cardiometabolic risk markers at 13.5 years by sex

	Boys (*n* = 259)		Girls (*n* = 286)		*p*
	Median	IQR	Median	IQR	
BMI (kg/m^2^)	16.4	(15.2, 18.0)	17.9	(16.2, 20.4)	<0.001
Overweight and obese^a^	16	(6.2%)	43	(15%)	0.001
Fat mass (kg)	6.3	(4.60, 9.00)	11	(8.40, 13.9)	<0.001
Fasting glucose (mmol/l)	5.06	(4.83, 5.33)	5.03	(4.78, 5.28)	0.06
Fasting insulin (pmol/l)	33.6	(21.8, 46.1)	45.5	(35.3, 62.0)	<0.001
Total cholesterol (mmol/l)^b^	3.5	(0.72)	3.56	(0.68)	0.26
Triglycerides (mmol/l)	0.69	(0.50, 0.98)	0.78	(0.59, 1.03)	0.005
HDL-cholesterol (mmol/l)^b^	1.09	(0.26)	1.05	(0.25)	0.1
LDL-cholesterol (mmol/l)^b^	2.04	(0.56)	2.12	(0.53)	0.11
HOMA-IR	1.24	(0.80, 1.76)	1.67	(1.27, 2.28)	<0.001
Systolic blood pressure (mmHg)^b^	110.9	(8.32)	108	(7.57)	<0.001
Diastolic blood pressure (mmHg)^b^	62.8	(6.9)	56.6	(6.74)	<0.001

^a^Values are number and percentage. Children were classified overweight or obese if their BMI-for-age, computed using the WHO growth standard, was greater than 1 SD. Differences between boys and girls are tested using Chi-squared test.

^b^Mean and standard deviation are reported for normally distributed variables. Differences between boys and girls are tested using two sample *t*-test and Mann–Whitney *U*-test for normally and not normally distributed variables, respectively.

Children with earlier BMI-derived AR had higher BMI (−0.58 per SD increase of BMI-derived AR; 95% CI [−0.65, −0.52]; *p* < 0.001) and fat mass (−0.44 per SD; 95% CI [−0.50, −0.37]; *p* < 0.001) at 13.5 years. Similar associations were obtained between skinfold-derived AR and BMI (−0.53 per SD increase of skinfold-derived AR; 95% CI [−0.61, −0.46 /^2^]; *p* < 0.001) and fat mass (−0.46 ; 95% CI [−0.53, −0.39]; *p* < 0.001) at 13.5 years. The relationships were not attenuated by adjusting for age, sex, height, socio-economic status and pubertal stage at 13.5 years, BMI or skinfold at time of rebound or maternal gestational diabetes.

Earlier BMI-derived AR was associated with lower HDL-cholesterol and higher fasting insulin, HOMA-IR and blood pressure (systolic and diastolic) (Table 2). Adjusting for age, sex, height, socio-economic status, pubertal stage at 13.5 years and maternal gestational diabetes attenuated the associations with diastolic blood pressure, but did not influence the other associations. The associations remained significant after adjusting for BMI at the time of rebound. After adjustment for fat mass at 13.5 years, all became non-significant (Table 2). Similar associations, in both direction and magnitude, were observed when considering skinfold-derived AR (Table 2).

**Table 2:** Coefficient estimates (and 95% confidence interval) for the regression of age at adiposity rebound on CVD risk factors at 13.5 years

	BMI-derived adiposity rebound (*z*-score)				Skinfold-derived adiposity rebound (*z*-score)			
	*Model 1* Estimate (95% CI)	*Model 2* Estimate (95% CI)	*Model 3* Estimate (95% CI)	*Model 4* Estimate (95% CI)	*Model 1* Estimate (95% CI)	*Model 2* Estimate (95% CI)	*Model 3* Estimate (95% CI)	*Model 4* Estimate (95% CI)
Fasting glucose	0.01	0.01	−0.03	−0.02	0.02	0.05	0.00	0.01
(SDS)	(−0.08, 0.09)	(−0.08, 0.11)	(−0.13, 0.07)	(−0.13, 0.08)	(−0.07, 0.11)	(−0.05, 0.14)	(−0.10, 0.11)	(−0.10, 0.12)
Fasting insulin	−0.21	−0.15	−0.13	0.02	−0.19	−0.14	−0.12	0.00
(SDS)	(−0.28, −0.13)^**^	(−0.23, −0.07)^**^	(−0.22, −0.04)^*^	(−0.07, 0.11)	(−0.27, −0.11)^**^	(−0.22, −0.05)^**^	(−0.22, −0.02)^*^	(−0.04, 0.15)
Total cholesterol	0.00	−0.04	−0.06	0.03	0.00	−0.05	−0.05	0.02
(SDS)	(−0.09, 0.08)	(−0.13, 0.05)	(−0.15, 0.04)	(−0.07, 0.13)	(−0.09, 0.09)	(−0.15, 0.04)	(−0.15, 0.06)	(−0.09, 0.12)
Triglycerides	−0.03	−0.05	−0.04	0.02	−0.01	−0.02	0.00	0.06
(SDS)	(−0.12, 0.05)	(−0.14, 0.04)	(−0.14, 0.05)	(−0.08, 0.12)	(−0.10, 0.07)	(−0.12, 0.08)	(−0.10, 0.11)	(−0.04, 0.17)
HDL-cholesterol	0.12	0.10	0.04	0.04	0.13	0.08	0.05	0.02
(SDS)	(0.04, 0.21)^*^	(0.00, 0.17)^*^	(−0.05, 0.14)	(−0.06, 0.14)	(0.04, 0.21)^*^	(−0.01, 0.18)	(−0.06, 0.15)	(−0.09, 0.12)
LDL-cholesterol	−0.04	−0.07	−0.07	0.02	−0.04	−0.1	−0.08	0.00
(SDS)	(−0.12, 0.04)	(−0.16, 0.02)	(−0.17, 0.02)	(−0.08, 0.12)	(−0.13, 0.05)	(−0.19, 0.00)	(−0.18, 0.03)	(−0.11, 0.11)
HOMA-IR	−0.2	−0.14	−0.13	0.02	−0.18	−0.12	−0.11	0.06
(SDS)	(−0.28, −0.12)^**^	(−0.22, −0.06)^**^	(−0.22, −0.04)^*^	(−0.07, 0.11)	(−0.26, −0.09)^**^	(−0.21, −0.03)^**^	(−0.21, −0.02)^*^	(−0.04, 0.15)
Systolic blood	−0.20	−0.14	−0.10	−0.07	−0.19	−0.13	−0.11	−0.06
pressure (SDS)	(−0.28, −0.11)^**^	(−0.23, −0.05)^*^	(−0.19, −0.01)^*^	(−0.17, 0.03)	(−0.28, −0.11)^**^	(−0.22, −0.04)^*^	(−0.21, 0.00)^*^	(−0.16, 0.05)
Diastolic blood	−0.11	−0.05	−0.05	−0.04	−0.08	−0.03	−0.03	−0.02
pressure (SDS)	(−0.19, −0.03)^*^	(−0.14, 0.05)	(−0.14, 0.05)	(−0.14, 0.06)	(−0.18, −0.01)^*^	(−0.13, 0.06)	(−0.13, 0.07)	(−0.13, 0.08)

Model 1 was adjusted for age at 13.5 and sex. Model 2 was adjusted for sex, age at 13.5, height, socio-economic status, pubertal stage, and maternal gestational diabetes.

Model 3 was further adjusted for BMI/skinfolds at the time of rebound. Model 4 was further adjusted for BMI or skinfold at time of rebound and fat mass at 13.5 years

*SDS* standard deviation score

**p-value < 0.001, *p-value < 0.05.

The associations between BMI-derived AR and systolic blood pressure, and those between skinfold-derived AR and fat mass, fasting insulin and HOMA-IR were in the same direction in boys and girls, but were stronger in boys (Figure S1). For these variables, significant positive interaction terms (*p* < 0.02) were observed.

### Sensitivity analysis: removing children with no rebound in adiposity

Of the 604 children in the study, 104 had a rebound in BMI but not in skinfolds, while 5 experienced neither a BMI-derived AR not a skinfold-derived AR. The majority (82%) of children who did not reach a skinfold rebound were girls; specifically, a rebound in skinfolds was not observed in 20 (7%) boys and 89 (29%) girls.

After removing the 109 children with no rebound in adiposity, mean (SD) BMI-derived age at AR was 6.22 (0.99) years for boys and 6.27 (1.09) years for girls (estimated difference: 0.05 years; 95% CI: [−0.12, 0.24]; *p* = 0.96), whereas mean (SD) skinfold-derived age at AR was 6.92 (1.10) years for boys and 6.31 (1.26) years for girls (estimated difference: −0.62 years, 95% CI: [−0.85, −0.41]; *p* < 0.001). As in the main analysis, boys reached skinfold-derived AR later than BMI-derived AR (estimated difference: 0.71 years; 95% CI: [0.58, 0.81]; *p* < 0.001); however, there was no significant difference between skinfold-derived AR and BMI-derived AR in girls (estimated difference: 0.04 years; 95% CI: [−0.11, 0.17]; *p* = 0.29). As observed in the full sample, there were significant associations of BMI-derived AR and skinfold-derived AR with cardio-metabolic risk markers measured in adolescence. The relationships were not attenuated by adjusting for age, sex, height, socio-economic status and pubertal stage at 13.5 years, BMI or skinfolds at time of rebound, or maternal gestational diabetes (Table S1). Similar significant interactions of sex with age at AR were observed (results not shown).

### Sensitivity analysis: removing children with incomplete measures of BMI or skinfolds

Four hundred and sixty children (76%) had complete measures of BMI and skinfolds between 2 and 10 years of age. Among those, 444 were followed up at 13.5 years. The percentages of boys and girls were the same observed in the full sample. After removing those with incomplete information on BMI and/or skinfolds we obtained results similar to those presented beforehand (results not shown).

## Discussion

This study showed that BMI and skinfolds have different growth patterns. Both increased rapidly in the first two post-natal years; BMI then decreased, reached a minimum around 5–7 years, and started increasing again; whereas skinfolds remained quite constant until 5–7 years and increased rapidly thereafter. The majority of children in the cohort had a rebound in skinfolds and BMI between 2 and 10 years of age; however, no skinfold rebound occurred for 18% of the children. Skinfold-derived AR differed significantly in age from BMI-derived AR. Associations between BMI-derived AR and skinfold-derived AR with later cardio-metabolic outcomes were similar in magnitude and direction, with coefficient’s estimates of BMI-derived AR lying within the 95% confidence interval of those of skinfold-derived AR. Overall, children with earlier AR had higher BMI, fat mass, fasting insulin, HOMA-IR and systolic blood pressure, and lower HDL-cholesterol at 13.5 years. The observed associations were independent of BMI/skinfolds at the time of rebound, but they were fully explained by fat mass at 13.5 years.

The average age at BMI-derived AR was comparable to that observed in European cohorts of children born between the 1930s and 1950s [8, 11]. Recent evidence suggests that, in Europe, the age at BMI-derived AR has fallen since the 1950s as a result of faster height gain in the first 2 years of life and advanced maturation (for example, indicated by earlier puberty) [8, 30]. In a Chilean study of children born in 2002–2003, 44% of children were less than 5 years old at BMI-derived AR [9]; however, the percentage of obese and overweight children was more than fourfold higher than in our cohort. There are not enough data to compare BMI-derived AR trends in India; however, a later BMI-derived AR (mean: 6.6 years, SD: 1.7 years) [31] observed in the New Delhi Birth Cohort (children born between 1969 and 1972) suggests that BMI-derived AR has fallen in India too.

Literature comparing BMI growth with that of more direct measures of adiposity is scarce. Collecting longitudinal adiposity data is challenging. Two commonly used techniques are bioelectrical impedance analysis and dual energy X-ray absorptiometry (DXA). The former is strongly influenced by individual characteristics (e.g. muscle mass), hydration and external temperature [32], whereas the latter is expensive, requires highly trained staff and involves radiation exposure, making it unsuited for the frequent serial measurements required to define AR precisely. A German cross-sectional study of more than 15,000 children, comparing the growth of BMI and fat mass index measured using bioimpedance, showed that BMI rebound occurred earlier than fat mass index rebound in both sexes [33]. We used longitudinal skinfold measures to characterise adiposity. Skinfold measures are quick, inexpensive, simple to obtain and, in school-aged children, are highly correlated with fat mass estimated using DXA [34]. We found that the majority of children reached a skinfold rebound between 2 and 10 years. However, the age at skinfold-derived AR differed from the age at BMI-derived AR. Boys reached skinfold-derived AR later than BMI-derived AR, whereas the opposite was observed in girls. Moreover, a third of the girls did not have a rebound in skinfolds suggesting that, in this population, BMI-derived AR does not represent a simple rebound in adiposity.

Regardless of sex and the measure used to identify AR, age at AR was inversely associated with BMI, fat mass and cardio-metabolic risk markers at 13.5 years. Similarly to findings in Western populations, these associations were independent of BMI at time of rebound [10]. Numerous studies have shown associations between age at BMI-derived AR and later cardio-metabolic risk markers [7, 9–11]. Others have compared the relative merits of cross-sectional measures of childhood/adolescent BMI and skinfolds at single time points in predicting body fat and later cardio-metabolic risk factors [35, 36]. However, few studies have compared associations of age at AR, estimated from longitudinal measures of adiposity, with cardio-metabolic risk markers in later life. One longitudinal study in the USA [37] found that children with earlier BMI rebound (≤5 years) or earlier triceps rebound (≤5 years) were more likely to be obese adults. Associations between AR and later cardio-metabolic risk markers were not reported.

In our study, associations between early AR and cardio-metabolic risk markers at 13.5 years were fully mediated by fat mass measured at the same age. This suggests the presence of an indirect pathway between AR and later risk outcomes, with children who rebound earlier having higher fat mass at 13.5, and consequently, higher fasting insulin, triglycerides, HOMA-IR and systolic blood pressure. Similar pathways were observed in younger Chilean children [9].

BMI and skinfold growth might help to identify children at risk of obesity, Type 2 diabetes and CVD in later life; however, they remain problematic to monitor and AR is rarely used in a clinical setting. Data on height and weight need to be collected frequently through childhood, and even if enough measures are available, the age at rebound can only be identified retrospectively. It has been suggested that BMI at age 7 could be a more feasible alternative to AR in assessing later obesity as correlations of BMI at age 7 with BMI measured at 18 and 21 years are similar in magnitude to those between AR and later BMI [38]. In our study, the correlation between BMI at age 7 and 13.5 was comparable in magnitude to that between BMI-derived AR and BMI at age 13.5 (results not shown). We are currently following up the cohort, and will be able to test whether this trend continues in early adulthood.

### Strengths and limitations

The availability of frequently and prospectively collected anthropometry constitutes a major strength of the study. Only 4% of the children had fewer than three measures of BMI and/or skinfolds; hence, it is unlikely that their exclusion affected the results. Complete information on BMI and skinfolds was available for 76% of the children. Sixty-four (10%) were missing values at only one time point, while 24 (4%) were missing values at 2 time points. Most missing values occurred before the age of 3 (*N* = 18 [3% of the total sample]) or after 8.5 (*N* = 48 [7% of the total sample]) and therefore are unlikely to bias the AR values. A sensitivity analysis, including only children with complete measures of BMI and skinfolds, showed that estimates of AR were not affected by missing values. Generalisability of the results constitutes an important limitation. We were only able to assess skinfolds as direct measures of adiposity, since bio-impedance data were first collected at the 5-year follow-up. Skinfolds only measure adipose tissue at one location; therefore, they might give different results from total body fat. Although different models were fitted and care was taken in choosing the appropriate confounders, other unmeasured variables (e.g. fat mass at time of rebound) could attenuate the observed associations.

## Conclusion

A rebound in adiposity as measured by skinfolds was detected in the majority of children. BMI-derived AR and skinfold-derived AR were different, thought the latter might contribute to the overall trend of BMI. Regardless of when BMI and skinfold rebound occur, they exhibited similar associations with obesity and cardio-metabolic risk factors at 13.5 years. These associations were not attenuated by BMI/skinfold at time of AR, but were fully explained by fat mass at 13.5 years. Although is not a true measure of adiposity, BMI rebound predicted later cardio-metabolic risk markers similarly to skinfold rebound. The ease of measurement makes BMI-derived AR a more achievable indicator in practice. Identifying the determinants of an early age at AR is the next essential step in understanding how to prevent later obesity and related disorders.

## Electronic supplementary material

Table S1

Figure S1
