# Supplementary material for: Comparing BMI with skinfolds to estimate age at adiposity rebound and its associations with cardio-metabolic risk markers in adolescence
Source: Int J Obes (Lond). 2018 Jul 13;43(4):683–90. doi: 10.1038/s41366-018-0144-8 (PMC6230257; doi:10.1038/s41366-018-0144-8)
Supplement: Supplementary file 1 — Table S1 [file 41366_2018_144_MOESM1_ESM.docx]

| **Table S1:** Coefficient estimates (and 95% confidence interval) for the regression of age at adiposity rebound on CVD risk factors at 13.5 years.  Sample comprised of children with known rebound and cardio-metabolic markers at 13.5 years (N = 451) | | | | | | | | | | | | | | | | | |
| --- | --- | --- | --- | --- | --- | --- | --- | --- | --- | --- | --- | --- | --- | --- | --- | --- | --- |
|  |  | **BMI- derived Adiposity Rebound** | | | | | | | |  | **Skinfold-derived Adiposity Rebound** | | | | | | |
|  |  | **(z-score)** | | | | | | | |  | **(z-score)** | | | | | | |
|  |  | *Model 1* |  | *Model 2* |  | *Model 3* |  | | *Model 4* |  | *Model 1* |  | *Model 2* |  | *Model 3* |  | *Model 4* |
|  |  | Estimate |  | Estimate |  | Estimate |  | | Estimate |  | Estimate |  | Estimate |  | Estimate |  | Estimate |
|  |  | (95% CI) |  | (95% CI) |  | (95% CI) |  |  | (95% CI) |  | (95% CI) |  | (95% CI) |  | (95% CI) |  | (95% CI) |
| BMI |  | -0.52 |  | -0.46 |  | -0.36 |  | - | |  | -0.53 |  | -0.48 |  | -0.44 |  | - |
| (SDS) |  | (-0.59, 0.44)^**^ |  | (-0.55, -0.39)^**^ |  | (-0.43, -0.29)^**^ |  | - | |  | (-0.61, -0.45) ^**^ |  | (-0.56, -0.39)^**^ |  | (-0.52, -0.36)^**^ |  | - |
| Fat mass |  | -0.37 |  | -0.33 |  | -0.26 |  | - | |  | -0.45 |  | -0.40 |  | -0.37 |  | - |
| (SDS) |  | (-0.45, -0.29) ^**^ |  | (-0.41, 0.25)^**^ |  | (-0.34, -0.19)^**^ |  | - | |  | (-0.52, -0.37) ^**^ |  | (-0.48, -0.32)^**^ |  | (-0.45, -0.29)^**^ |  | - |
| Fasting glucose |  | 0.00 |  | -0.01 |  | -0.03 |  | -0.01 | |  | 0.04 |  | 0.05 |  | 0.04 |  | 0.04 |
| (SDS) |  | (-0.10, 0.09) |  | (-0.10, 0.09) |  | (-0.13, 0.07) |  | (-0.12, 0.09) | |  | (-0.06, 0.14) |  | (-0.05, 0.16) |  | (-0.07, 0.14) |  | (-0.08, 0.16) |
| Fasting insulin |  | -0.18 |  | -0.15 |  | -0.12 |  | -0.01 | |  | -0.29 |  | -0.24 |  | -0.22 |  | -0.07 |
| (SDS) |  | (-0.27, -0.10)^**^ |  | (-0.23, -0.05)^**^ |  | (-0.22, -0.03)^**^ |  | (-0.10, 0.08) | |  | (-0.38, -0.21)^**^ |  | (-0.33, -0.14)^**^ |  | (-0.32, -0.14)^**^ |  | (-0.17, 0.03) |
| Total cholesterol |  | 0.03 |  | -0.01 |  | -0.03 |  | 0.04 | |  | 0.03 |  | -0.03 |  | -0.04 |  | 0.04 |
| (SDS) |  | (-0.06, 0.12) |  | (-0.11, 0.09) |  | (-0.13, 0.07) |  | (-0.06, 0.15) | |  | (-0.07, 0.12) |  | (-0.13, 0.07) |  | (-0.13, 0.07) |  | (-0.07, 0.16) |
| Triglycerides |  | 0.03 |  | 0.01 |  | 0.02 |  | 0.05 | |  | 0.01 |  | -0.01 |  | 0.00 |  | 0.04 |
| (SDS) |  | (-0.06, 0.12) |  | (-0.09, 0.10) |  | (-0.08, 0.12) |  | (-0.06, 0.15) | |  | (-0.09, 0.11) |  | (-0.11, 0.09) |  | (-0.11, 0.10) |  | (-0.07, 0.16) |
| HDL-cholesterol |  | 0.10 |  | 0.08 |  | 0.04 |  | 0.03 | |  | 0.11 |  | 0.08 |  | 0.07 |  | 0.03 |
| (SDS) |  | (0.01, 0.19)^*^ |  | (-0.02, 0.17) |  | (-0.06, 0.14) |  | (-0.06, 0.14) | |  | (0.02, 0.21)^*^ |  | (-0.02, 0.18) |  | (-0.03, 0.17) |  | (-0.08, 0.15) |
| LDL-cholesterol |  | -0.02 |  | -0.05 |  | -0.06 |  | 0.02 | |  | -0.03 |  | -0.08 |  | -0.07 |  | 0.03 |
| (SDS) |  | (-0.11, 0.07) |  | (-0.15, 0.04) |  | (-0.16, 0.04) |  | (-0.08, 0.12) | |  | (-0.12, 0.07) |  | (-0.18, 0.02) |  | (-0.17, 0.04) |  | (-0.09, 0.14) |
| HOMA-IR |  | -0.17 |  | -0.13 |  | -0.12 |  | -0.03 | |  | -0.27 |  | -0.22 |  | -0.21 |  | -0.06 |
| (SDS) |  | (-0.26, -0.09)^**^ |  | (-0.23, -0.04)^**^ |  | (-0.22, -0.03)^*^ |  | (-0.13, 0.07) | |  | (-0.36, -0.18)^**^ |  | (-0.31, -0.12)^**^ |  | (-0.31, -0.12)^**^ |  | (-0.16, 0.04) |
| Systolic Blood Pressure |  | -0.21 |  | -0.16 |  | -0.14 |  | -0.10 | |  | -0.25 |  | -0.22 |  | -0.22 |  | -0.16 |
| (SDS) |  | (-0.29, -0.12)^**^ |  | (-0.25, -0.07)^*^ |  | (-0.24, -0.05)^*^ |  | (-0.20,0.00)^*^ | |  | (-0.34, -0.16)^**^ |  | (-0.32, -0.12)^**^ |  | (-0.32, -0.12)^**^ |  | (-0.27, -0.06)^**^ |
| Diastolic Blood Pressure |  | -0.08 |  | -0.04 |  | -0.04 |  | -0.03 | |  | -0.05 |  | -0.02 |  | -0.03 |  | -0.01 |
| (SDS) |  | (-0.17, 0.01) |  | (-0.13, 0.06) |  | (-0.14, 0.06) |  | | (-0.13, 0.07) |  | (-0.14, 0.05) |  | (-0.12, 0.07) |  | (-0.13, 0.07) |  | (-0.16, 0.11) |
| Model 1 was adjusted for age at 13.5 and sex. Model 2 was adjusted for sex, age at 13.5, height, socio-economic status, pubertal stage, and maternal gestational diabetes.  Model 3 was further adjusted for BMI/skinfolds at the time of rebound. Model 4 was further adjusted for BMI or skinfold at time of rebound and fat mass at 13.5 years. SDS, standard deviation score.  ^**^p-value < 0.001, ^*^p-value < 0.05. | | | | | | | | | | | | | | | | | |
|  |  |  |  |  |  |  |  |  |  |  |  |  |  |  |  |  |  |
|  |  |  |  |  |  |  |  |  |  |  |  |  |  |  |  |  |  |
