# Supplementary material for: Comparing BMI with skinfolds to estimate age at adiposity rebound and its associations with cardio-metabolic risk markers in adolescence
Source: Int J Obes (Lond). 2018 Jul 13;43(4):683–90. doi: 10.1038/s41366-018-0144-8 (PMC6230257; doi:10.1038/s41366-018-0144-8)
Supplement: Supplementary file 2 — Figure S1 [file 41366_2018_144_MOESM2_ESM.docx]

**Figure S1:** Interaction plots of adiposity rebound and sex for selected outcomes. (a) refers to the association between BMI-derived adiposity rebound and systolic blood pressure according to sex. (b), (c) and (d) refer to the associations of skinfold-derived adiposity rebound with fat mass, HOMA-IR and fasting insulin respectively. All cardio-metabolic markers were measured at 13.5 years. P-values for the interaction term are reported within each plot.


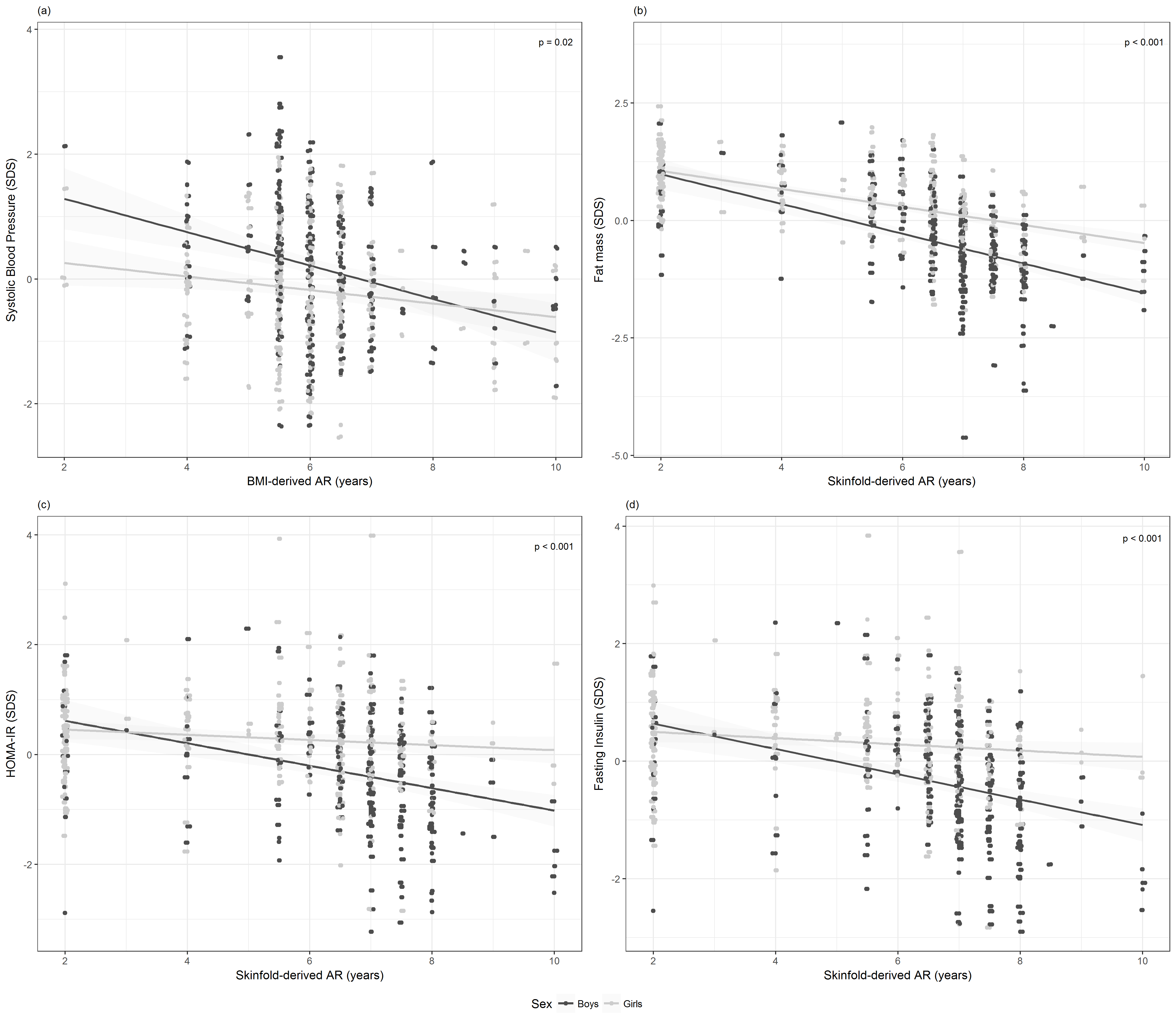


AR, adiposity rebound. SDS, standard deviation score.
